# Data on the relationship between bromide content and the formation potential of THMs, HAAs, and HANs upon chlorination and monochloramination of Karoon River water, Iran

**DOI:** 10.1016/j.dib.2016.05.068

**Published:** 2016-06-02

**Authors:** Samad Akbarzadeh, Raheleh Kafaei, Seyedenayat Hashemi, Bahman Ramavandi

**Affiliations:** a Department of Biochemistry, The Persian Gulf Biotechnology Research Center, Bushehr University of Medical Sciences, Bushehr, Iran; b Environmental Health Engineering Department, Faculty of Health, Bushehr University of Medical Sciences, Bushehr, Iran

**Keywords:** Chlorination, Monochloramination, Bromide ion, Disinfection by products, Karoon River

## Abstract

This data article reports the relationship between of the bromide ion concentration and the formation potential of disinfectant byproducts (DBPs) including, trihalomethanes (THMs), haloacetic acids (HAAs), and haloacetonitriles (HANs) upon chlorination and monochloramination of the raw water of Karoon River water in Iran. Water samples were collected at an intake of a drinking water treatment plant during July 2014. All tests were performed in triplicate (*n*=3) and the mean of three measurements reported herein. The data of the formation potential of DBPs was determined under different bromide ions content. The data show the relationship between bromide concentration and DBPs formation that will be useful in the future management, operation and design of water treatment plants.

**Specifications Table**

**Table:** 

Subject area	Environmental Engineering
More specific subject area	Environmental assessment
Type of data	Table
How data was acquired	Four species of THMs were analyzed using MTBE extraction–GC–ECD method.
	Nine species of HAAs were determined by MTBE extraction–acid methanol methylation–GC–ECD method.
	The concentration of HANs species was measured using a gas chromatograph with an electron capture detector.
	The chlorine and monochloramine were determined by the N,N diethyl-p-phenylenediamine (DPD) titrimetric method.
Data format	Analyzed
Experimental factors	A sample of Karoon River water was prepared from a water intake of a water treatment plant.
	Bromide ion content of Karoon River water was spiked to given values.
Experimental features	Relation of Br^−^ ion and disinfection by products speciation
Data source location	Bushehr University of Medical Sciences, Bushehr, Iran, GPS: 28.9667°N, 50.8333°E
Data accessibility	Data are available with the article

## Value of the data

• The data provides details on which strategy (chlorination and monochloramination) will be effective on the lowering of brominated-DBPs as most dangerous DBPs.
• The data will be beneficial for minimizing the DBPs formation and for an optimal designating of the disinfection plant in water treatment plants.
• This data article directly assesses what impact the switch from chlorine to monochloramine would have on the concentrations of the DBPs found; thus it will be useful in the future management and operation of water treatment plants.

## 1 Data

Table 1 is presented the chlorine and monochloramine residue as function of the disinfectant types (*i.e.*, chlorination and monochloramination). The data regarding to the relation of bromide ion content and THMs, HAAs, and HANs species upon chlorination and monochloramination of Karoon River water were depicted in Table 2, Table 3, Table 4, respectively.

## 2 Experimental design, materials and methods

### 2.1 Water sampling

Water sampling was done in July 2014 at a water intake in Karoon River where the water is pumped to the Mahshahr water treatment plant. About ten liters of water were collected at the water intake from a depth of approximately 50 cm below the water surface with a hand-held open-mouth bottle. The samples were placed in a cooler on ice, shipped to a laboratory within the same day, and stored at 4 °C before further test. Two liter of water was passed through a glass fiber filter (0.42 μm) to analysis the initial content of bromide ion of water samples.

### 2.2 Experimental procedure of chlorination/chloramination test

A stock of free chlorine (HOCl) solution was prepared by 5% sodium hypochlorite (NaOCl) (Sigma-Aldrich) and dilution to 1000 mg/L as Cl_2_. The prepared solution was stored in an aluminum foil-covered glass stoppered flask for further tests. The stock 5% NaOCl was purchased from Sigma-Aldrich. The monochloramine was obtained daily by mixing a given volume of NH_4_Cl and NaOCl solutions (Cl_2_/N ratio was 4/1 w/w) and the mixture was then incubated for 0.5 h in the dark. The monochloramine and chlorine were weekly standardized by the N,N diethyl-p-phenylenediamine (DPD) titrimetric method before disinfection test, and then, the known dosage of disinfectant solution was added to the bottles. The batch experimental runs were conducted in 100-mL amber bottles with PTFE–lined screw cap. After regulation the bromide ion content of the samples, a specified chlorine dose was poured to the water samples and then well–mixed at 120 rpm using a shaker incubator (Parsazma, Iran) under anaerobic conditions. Upon expire the reaction time, the residue of Cl_2_ and NH_2_Cl and disinfection by products (THMs, HAAs, and HANs) were determined under various Br^−^ concentrations (10, 100, 200, and 400 µg/L). All samples were done in triplicate (n= 3) to minimize the error from the experimental procedure.

### 2.3 Measurements

The residual concentrations of monochloramine and chlorine were determined by using a Hanna photometer HI-93711,based on DPD/FAS titration according to method presented in standard Methods for the Examination of Water and Wastewater (method number: 4500-Cl-G) [1]. THMs analyses were performed using a gas chromatography (GC) with an electron capture detector (ECD; series 6890 Agilent with DB 624 column, J&W Science), based on EPA method 551.1. The concentration of nine HAAs species including, CAA, BAA, DCAA, TCAA, BCAA, DBAA, BDCAA, DBCAA, and TBAA was measured by liquid– liquid extraction with methyl-tertiary–butyl–ether (MTBE) followed by derivatization with acidic methanol and by GC–ECD according to a modified version of EPA method 552.2 [1]. HANs including, DCAN, TCAN, BCAN, and DBAN analyses were carried out using a GC (Finnigan TRACE GC) with an electron capture detector [2]. Further details of THMs, HAAs, and HANs measurements including, continuing calibration check, detection limits, quality assurance–quality control, and recovery percentage are available elsewhere [3], [4], [5].

## Figures and Tables

**Table 1:** Chlorine and monochloramine residue as function of the disinfection conditions.

Parameters	Amount	Residue Cl_2_ (mg/L)	Residue NH_2_Cl (mg/L)
Contact time (h)	2	1.03	3.56
	24	0.88	3.32
	72	0.22	2.76
			
Cl_2_/NH_2_Cl^a^	Low	0.0	1.32
	Middle	1.1	3.22
	High	2.1	6.21
			
Br^−^ (µg/L)	10	1.02	3.32
	100	0.53	3.31
	200	0.54	3.01
	400	0.54	3.32
			
pH	6	0.67	2.35
	7	1.1	3.26
	8	0.53	3.54
			
Temperature (°C)	10	1.1	3.24
	20	0.96	3.25
	30	0.54	3.47

a Low means 0.6 mg/L Cl_2_ or 1.3 mg/L NH_2_Cl; middle means 1.8 mg/L Cl_2_ or 3.6 mg/L NH_2_Cl; high means 3.6 mg/L Cl_2_ or 7.4 mg/L NH_2_Cl.

**Table 2:** THMs values as function of bromide content upon chlorination and monochloramination of Karoon River water.

Br^−^ (µg/L)	Cl_2_				NH_2_Cl			
	CFM	BDCM	DBCM	BFM	CFM	BDCM	DBCM	BFM
10	11.11	0.88	0.12	0.01	1.00	0.02	0.01	0.01
100	5.71	3.79	5.04	1.22	0.91	0.19	0.09	0.04
200	3.65	4.47	9.61	4.49	0.89	0.32	0.24	0.12
400	2.09	3.42	11.36	12.35	0.99	0.46	0.55	0.54

Unit of THMs: µg/L

Chloroform (CFM), bromodichloromethane (BDCM), dibromochloromethane (DBCM), and bromoform (BFM).

**Table 3:** HAAs values as function of bromide content upon chlorination and monochloramination of Karoon River water.

Disinfectant	Br^−^ (µg/L)	CAA	BAA	DCAA	TCAA	BCAA	DBAA	BDCAA	DBCAA	TBAA
Cl_2_	10	0.40	0.06	12.02	11.23	1.06	0.07	1.90	0.17	0.00
	100	ND	0.44	7.17	3.45	4.19	2.75	7.39	5.23	2.21
	200	ND	0.69	4.70	1.99	4.85	5.58	7.73	9.96	6.55
	400	ND	0.96	2.33	0.79	3.78	7.63	4.19	9.40	9.68
										
NH_2_Cl	10	0.89	0.01	3.07	0.61	0.23	ND	ND	0.24	ND
	100	ND	0.04	2.41	0.44	1.23	0.69	0.03	0.28	0.26
	200	ND	0.09	1.94	0.43	1.49	1.54	0.06	0.11	0.75
	400	ND	0.14	1.72	0.41	1.81	3.15	0.08	0.20	1.68

ND: Non-detectable.

Unit of HAAs: µg/L.

Chloroacetic acid (CAA), dichloroacetic acid (DCAA), trichloroacetic acid (TCAA), bromoacetic acid (BAA), bromochloroacetic acid (BCAA), dibromoacetic acid (DBAA), dibromochloroacetic acid (DBCAA), bromodichloroacetic acid (BDCAA) and tribromo acetic acid (TBAA).

**Table 4:** HANs values as function of bromide content upon chlorination and monochloramination of Karoon River water.

Disinfectant	Br^−^ (µg/L)	DCAN	TCAN	BCAN	DBAN
Cl_2_	10	0.02	0.29	ND	ND
	100	0.01	0.01	ND	0.16
	200	0.01	ND	ND	0.36
	400	0.01	ND	ND	0.51
					
NH_2_Cl	10	0.01	0.08	ND	ND
	100	0.01	0.02	0.05	0.04
	200	0.01	0.01	0.04	0.05
	400	0.01	0.01	0.04	0.09

ND: Non-detectable.

Unit of HANs: µg/L.

Dichloroacetonitrile (DCAN), bromochloroacetonitrile (BCAN), dibromoacetonitrile (DBAN), and trichloroacetonitrile (TCAN).
